# Evidence-based policy-making - epidemiology as a key science for quality of life in society

**DOI:** 10.1007/s10654-023-01056-7

**Published:** 2023-11-08

**Authors:** Karl W. Lauterbach

**Affiliations:** Minister of Health, Federal Ministry of Health, 11055 Berlin, Germany

**Keywords:** Evidence-based policy-making, Epidemiology, Ageing, Burden of disease, Pandemics, Climate change, Zoonoses, Environmental health, Epidemics, Emerging infectious diseases

## Introduction

I was very honoured to be invited to present my insights into evidence-based policy-making in the European Journal of Epidemiology, based on the 174th Cutter lecture I delivered at Harvard T.H. Chan School of Public Health. Having worked with a variety of competent and dedicated colleagues and motivated students over the past years, both as a trained medical doctor and clinical epidemiologist and as the German Minister of Health, I am pleased to see a vast increase in awareness of the importance of epidemiology and of policy-making based on scientific evidence. Here I will try to share some experiences and points for discussion, most of which were also included in my Cutter lecture.

### Early experiences

To enter Harvard School of Public Health more than 30 years ago was not an easy decision for me. Initially, I had wanted to become a cardiothoracic surgeon. However, it already occurred to me as a fourth-year medical student, when working in the cardiothoracic operating room at the San Antonio Health Sciences Center in Texas, that 80 or 90% of patients at that time appeared to be suffering from diseases or injuries that could have been prevented. Thus, torn between helping individual patients on the one hand, and conducting epidemiological research and making that research available to a wider population on the other, was how I came to make up my mind to leave surgery and to proceed with epidemiology through the MPH programme at Harvard Medical School of Public Health. My initial advisor was Walter Willett. I later switched to the health policy department, where Marc Roberts became my advisor and mentor. After returning to Germany from the US, I worked to combine clinical epidemiology and health economics at the University of Cologne, where I founded an institute combining these research agendas.

Moving forward in my career, I became more ambitious about making what we know in science available to more people, rather than mainly conducting primary research. Therefore, after 10 years of research and advising politicians, I went into politics myself and became a member of the German Bundestag in 2005.

### Current and future challenges

I have always been convinced that one needs to know the science in order to effectively implement it and that it is imperative to improve evidence-based policy-making to make our health care systems more efficient. When I entered into politics, initially as a government advisor, we were still working on very localised, technical problems. For example, as advisor to then Minister of Health Ulla Schmidt, we knew that in Germany, average length of stay in hospitals was longer than medically necessary and that it was longer in Germany than in most other European countries [1]. So we introduced the German Diagnosis-Related Groups (G-DRG) system as a replacement for per diem payments in order to shorten lengths of stay [2]. And indeed, average length of stay in hospitals shortened extensively [1].

Since then, the DRG system in Germany has led to secondary effects that make it necessary to once more move forward and change the hospital payment system again. These DRGs are having a negative impact on evidence-based treatment decisions in Germany, because they create strong incentives for overtreatment and cost-cutting. Therefore, the comprehensive hospital payment reform currently underway in Germany would see 60% of funds come from service maintenance flat rates (“*Vorhaltepauschale”*) to ensure available healthcare capacities and only 40% from actual patient treatment. This reform is based on extensive research by a government commission of 17 scientists serving as official hospital-reform advisors in my ministry. At the time of writing, they have been working for 14 months on preparing this much-needed reform.

Another example is the enactment of a policy to improve the quality of medical procedures through minimum volume laws, e.g. for pancreatic cancer surgery or joint replacements. In Germany, these procedures can only be billed by a provider if they perform a certain minimum number of procedures every year. The law was largely inspired by the pioneering work of John Birkmeyer [3, 4].

A third example of evidence-based policy-making is diabetes treatment in Germany. In the late 90s, diabetes tended to be over-treated, by focusing almost exclusively on lowering HBA-1 C levels. However, the research at that time clearly showed that especially in elderly patients, blood pressure control, which was usually under-treated, should be another priority [5]. Therefore, disease management programmes in primary care (DMPs) were introduced in Germany, implementing evidence-based guidelines for two medical conditions in 2003. The programmes have since been extended and are updated regularly. In all of these reforms, before I became a member of parliament, I was involved as a government advisor in a team of other scientists, all committed to the endeavour to bringing scientific evidence into policy-making. Not all of the laws passed fulfilled their objectives, but all were at least evaluated, their evaluation having been legally required and improvements were attempted.

Eventually, between the late 90s and 2005, which was when I became a member of parliament, I was doing more or less exactly what I had learned at Harvard School of Public Health, putting evidence into health policy. It is clear that this approach is not always successful and that political experience is a key factor in predicting how even sound, evidence-based policies may fail or cause unintended consequences, something I had to find out the hard way. I also learned that often what may have worked in the past, needs to change if the initial idea has been corrupted by negative incentives. Most importantly, I learned that excessive financial incentives in health care can corrupt evidence-based medicine. As I previously mentioned, we are now in the process of developing a different policy on how to reimburse hospitals in the future. So we will now move forward, beyond DRGs. But, once again, we will do so based on evidence-based policy-making rules, since any other alternatives would be worse.

### New extraordinary challenges

In the following, I will focus on three extraordinary challenges that go well beyond the scope of the localised problems in need of the technical fixes mentioned above. We will not master these challenges unless there is a major change in how we approach things more generally. As a policymaker, as a member of parliament and now as a minister, I have oftentimes worked on very specific problems where there was scientific consensus, where policymakers knew what was needed and we could work on bringing science and politics into alignment. With all these policy decisions, delay obviously meant tragedy for those individuals who did not live to see the day progress was made. For them, such progress came too late. However, there are now major problems of a different nature. Firstly, it is no longer individuals, but entire generations that are at risk. Secondly, the gap between what is known to solve the problems we face and what we are currently doing is much wider than in standard health policy-making. In my mind, the three main challenges of our time that require the most stringent and urgent evidence-based policy-making are as follows: ageing, climate change and the protection of the environment.

## Ageing

### Global demographic transformation

The challenge of the baby boomer generation with regards to ageing is vastly under-recognised, not just in the most industrialised countries. Globally, the proportion of elderly people is growing at a rapid pace. Currently, Europe has the highest percentage of population aged 60 or over (25%), surpassed in particular by Japan. However, rapid ageing will occur in other parts of the world as well. By 2050, people aged 60 years or over will account for about 25% or more of the population in all regions of the world, with the exception of Africa [6]. As a result, we will face major challenges in the healthcare and retirement sectors.

### Ageing of baby boomers

Currently, the baby boomer generation, is at the height of its economic productivity and is still reasonably healthy. In Europe, it is a reasonable estimate that more than one third of baby boomers already have at least one chronic condition. For example, a survey in Germany found that 38% of respondents suffer from at least one chronic condition [7]. In the US, where baby boomers are 5–10 years older than in Europe, the proportion is likely much higher. It is quite probable that some of them are not yet suffering from the complications of their chronic condition and that they do not yet have multiple chronic conditions. However, 10 to 15 years from now, this group of people will largely no longer be economically productive and will be almost certain to have a high morbidity. So as far as the health system is concerned, this group will basically switch sides. While currently often still being important providers of medical care and contributors to medical insurance, soon they will largely only be recipients of medical care. While now often the most economically productive members of society, in future they will be the recipients of retirement benefits. That change has to be noted. Germany and the US face additional problems, since their life expectancy has developed at a slower pace than northern or southern European countries, even more so in comparison to Japan. Very high expenses, mediocre outcomes and a lack of medical staff even now are a very poor baseline for addressing the ageing of the baby boomer generation indeed.

### The world is ageing each country at its own pace

Population ageing can also be expected in other countries, but different countries and regions of the world are ageing at their own pace.

By 2040, the United States is expected to have 22.4% of its population aged 65 and older, which corresponds to the same age group’s current proportion within the German population [8]. Thus, to some extent, the health impact of ageing on the socioeconomic system currently seen in Germany could also be expected in the US. What does that mean in practice? Well, in Germany we already spend around 40% of wages on social security and, on top of that, taxes. German society has thus far been fairly willing to support such costs through redistribution. However, not all countries are prepared to do this – which will also include Germany, if costs were to continue to spiral.

To this day, it has been one of my greatest challenges as Minister of Health to prevent the biggest ever deficit of the German health insurance funds. Without reform, the German health insurance funds were expected to run a 6% deficit of 17 billion euros in 2023. Painful cuts were demanded to balance the budget. How will we solve such problems in 10 years from now? We may have to decide: do we cut services or benefits, do we underprovide, should there be more co-payments or will there be more money from tax revenue or social security contributions? Germany ultimately decided against cuts and in favour of higher social security contribution rates. But how many times will that be possible? As of writing, another increase in the social security rates is next to unavoidable. Cutting services is unwise, because the benefit package is already based on proven needs in Germany. Hospital, primary care and drug pricing laws are on the way to make the system more efficient. Some of these laws are known to have been needed for 10 years. But these laws will only work in the future. Cutting benefits, simply because politics delayed clearly needed reforms, would appear ethically indefensible and politically unwise.

In Germany, population ageing will continue to proceed. By 2040, Germany is expected to have close to 30% of the population aged 65 and older [8]. This is comparable to the current proportion in Japan [8], where the situation is even more grave, with Japanese productivity already suffering because of the major challenges to its social security system. Since many highly developed countries are ageing at the same time, the global economic and social consequences are very difficult to predict.

### Socio-economic inequalities in longevity

Additionally, we often under-recognise the large inequality in longevity. The longevity distribution between countries does not reflect the inequity in terms of quality of life and life expectancy differences between the rich and the poor within countries. As an example, when comparing lowest and highest income groups in Germany, the mean life expectancy at birth shows a difference of almost 9 years for men and more than 4 years for women [9]. This trend can be seen in many other countries as well. In the US, the disparities are even greater, with a difference in life expectancy when comparing the richest and poorest 1% of individuals of almost 15 years for men and 10 years for women [10]. Similarly, in Norway, a difference in life expectancy between the richest and poorest 1% of individuals of almost 14 years for men and more than 8 years for women was reported [11]. So we have to face not only the ageing of the baby boomer generation, but also the unsolved problem of inequalities in life expectancy due to socio-economic status [12]. Lifestyle differences, a lack of preventive behaviour and worse access to high-quality medical care have proven to be much more entrenched than expected. Germany has made very little progress in addressing these inequalities, despite a number of laws targeted at improving equality of care, including comprehensive primary prevention interventions by the health insurance funds, disease management programmes, public hospital quality records and minimum volume requirements for public hospitals.

### Ageing-associated trends in burden of disease

On the topic of burden of disease, it is important to consider which diseases will become more important and which diseases become less important in terms of quality of life and survival as societies age [13]. With today’s knowledge, more than 80% of cardiovascular diseases are preventable under ideal conditions [14]. That may imply that other competing diseases will fill the gap when cardiovascular diseases wane. Indeed, cardiovascular diseases are gradually on the retreat. Unfortunately, it is a different issue when it comes to cancer and dementia, where currently we can at best prevent about 40% of cases of each [15, 16]. What does this mean for policy-making? When infectious diseases and cardiovascular diseases are outcompeted, it means that the majority of the baby boomers are, at some point in their lives, likely to suffer from cancer or from cognitive decline or dementia.

This may make medical care even more expensive. Let us take a look at the costs, for cancer treatment, for example: we are currently only using CAR-T-Cell therapy in a small number of lymphoma and leukaemia types, rarely for common solid tumours. However, it is likely that in the near future, we will be able to use CAR-T-Cell or other forms of personalised medicine for most if not almost all solid end-stage tumours. According to some estimates, this would entail enormous costs of 320,000 euros per treatment. If people survive the cancer, the likelihood of them getting dementia often even increases. In addition to the absolute numbers increasing due to higher survival rates, cancer and its treatment constitute another risk factor for later dementia [17].

A recent study in Germany further highlights the importance of specialised treatment facilities [18]. For ten out of eleven types of cancers analysed, there was a clear-cut advantage in terms of mortality for patients treated in certified cancer centres, compared to non-certified hospitals. Nevertheless, this knowledge is not yet adequately put into practice. For example, this could be addressed by having more certified centres for cancer treatment or by developing pathways to organise treatment outside the centres in the same way. Both goals are part of the hospital reform in Germany mentioned above.

### Impact of the COVID pandemic on life expectancy

Socio-economic background was a major driver of COVID mortality in Germany, echoing what is known for chronic diseases. And again, evidence-based treatment and specialisation were linked to better outcomes. For example, in Europe we can see a difference in terms of mortality if we compare ECMO treatment for interstitial lung disease for COVID at specialised centres and non-specialised centres. The mortality rates in specialised centres were much lower. For instance, a French study [19] reported a strong association of improved 90-day survival with increased experience of medical centres in performing ECMO, thus highlighting the relevance of evidence-based analyses and the implementation of evidence-based policies.

## Climate change

A second field where evidence-based policy-making urgently needs to be put into practice is climate change. A substantial body of data suggests that the cost of climate change will not only concern environmental issues, but will affect the individual more directly with respect to living conditions. When the imminent challenges of climate change are discussed, the connection to illness and a reduced quality of life are often not highlighted in the general discourse. However, it is fundamentally important to note what will happen to human mortality and quality of life when the climate drastically changes. Such a dramatic change is rather likely. Roughly speaking, we are currently moving towards what is called the shared socioeconomic pathway (SSP) 2-4.5 trajectory [20]. That means it is projected that we are likely heading towards a more than 2 °C increase in global warming, even under realistic as opposed to pessimistic modelling circumstances.

What does that mean for our health? In medical care, many cases – for example in cancer treatment – have tipping points. Once the cancer has spread, a cure is difficult, often impossible. Similarly, the problem with climate change is that now we are approaching tipping points, changes in our environment that interact in ways that mutually reinforce the speed and direction of global warming, affecting planetary liveability. We may come to a point where we can treat, but no longer “save the patient”. Once we go beyond tipping points, such as the Antarctic meltdown, boreal permafrost thaw or rainforest degradation the process may accelerate, pushing yet other tipping points to where it becomes almost impossible to reverse the overall situation.

To give you one example, in a 2 to 3 °C scenario, let’s call this a 2.5 °C median scenario, data suggests that the collapsing of the Greenland Ice Sheet might become one of the five instances of irreversible damage to our ecosystem. Here, the interactions between rising temperatures and reflection angles of the sun lead to so-called ice-albedo feedback, aggravating the problem of global warming in these areas to an uncontrollable scale.

The other four scenarios – the Labrador Sea convection collapse, the West Antarctic Ice Sheet collapse, the widespread abrupt permafrost thaw and the massive die-off of tropical coral reefs – are also all likely scenarios we will probably not be able to prevent [21, 22].

### The ten most important short-term steps to limit warming to 1.5 °C

Therefore, we need to get pragmatic. We have to go further than engage in abstract discussions in the climate debate and move towards creating and implementing more evidence-based policies. The kind of evidence-based policies that might, for example, be monitored by the climate action tracker. This consortium created by NGO groups of scientists has focused on the most important short-term measures we have to take to limit temperature rise to 1.5 °C [22].

According to their estimations, we have to renovate 3–5% of buildings every year. We need more zero-emission-buildings. We have to implement best practice in the field of agriculture. We have to stop deforestation and we have to sustain the growth of renewable energy sources as quickly as possible. We must further refrain from building new power plants. Fossil fuel-cars should no longer be sold after 2035. Moreover, we need technological advancements in the industry sector to reduce carbon emissions. If you want to see how much progress countries have made so far, you simply need to follow the climate-action record shown in the climate action tracker [23]. In their estimation, none of the countries they have assessed is currently on track to achieve the 1.5 °C climate change goals. Everyone is in favour of saving the climate. Everyone is in favour of lowering greenhouse gas production. Everyone basically knows what is needed. Nevertheless, when it comes to making a difference and taking action, most of us fall short.

### Health impacts of climate change

The health impacts of climate change are also vastly under-recognised. With climate change, we will have more extreme weather conditions. We will have extreme droughts, extreme rainfall, we will have extreme famines and so forth. This will lead to more injuries, more fatalities and more negative mental health effects. Heat stress immediately lowers the quality of life.

It is projected that globally we will lose major inhabited areas to climate effects [20]. People will have to use exponentially growing and increasingly economically unmaintainable measures to mitigate these effects in such areas. Some areas will become uninhabitable. Malnutrition or under-nutrition are predicted to spread and intensify. In some areas, the ecology as a whole is changing. This facilitates the spread of vectors such as mosquitoes or ticks, leading to a rise of vector-borne diseases. At the same time, a scarcity in resources such as water can lead to water-borne diseases. In this scenario, the reasons for rising conflicts and forced migration globally becomes logical and extremely feasible. Ultimately, all this is about how well we will be able to live.

Another problem is that the greatest climate changes are actually not expected to happen in the countries and continents with the highest carbon emissions. Europe and North America will certainly suffer from some effects of climate change that should not be underestimated, but the major brunt will have to be borne by mostly low-income countries [24]. In the COP27 meetings, a major stumbling block remained the failure to reach a consensus on financial support for these low-income countries to upscale their climate mitigation efforts. Still, the risk remains much too one-sided.

### Viral spread, zoonotic diseases and the impact on health

On the topic of climate change and pandemics: pandemics are not mandatory. The famous epidemiologist Billington said that outbreaks are mandatory, pandemics are optional. Therefore, if you manage an outbreak quickly enough, it will not become a pandemic. Even today, more than 60% of all human infectious diseases are of zoonotic origin, with 75% of emerging human infectious diseases currently having an animal origin [25]. Current climate research shows the risk of emerging zoonotic diseases is even growing due to climate change, as the interactions of humans and unexplored wildlife and habitats increases [26, 27].

Therefore, we can expect more zoonotic diseases to emerge, meaning that we can also expect more pandemics. The global data trajectory shows an increase, for example in dengue fever. Studies conducted in the highlands of North and South America and Africa suggest an increase in the number of months during which malaria can be transmitted. So tropical diseases have a chance to spread to what are currently more temperate climate zones and, in some cases, are already at our doorstep.

### Land use and promoting plant-based diets

With regard to hunger and malnutrition, it is important to note that most of the land currently used for food production, is actually used to produce animal feed, while only 14% is used for food for human consumption. From my perspective: present-day hunger is exclusively man-made. There is no need for anyone to go hungry nowadays.

So we ask ourselves: What is the role of epidemiology in this situation? Data suggests that switching to plant-based diets reduces not only greenhouse emissions, but also reduces health-related risks [28]. Meaning you could make people healthier and reduce greenhouse emissions at the same time.

## Environmental Health

Now I come to the third part, where epidemiology is a key science for focused policy-making.

### Air pollution – the silent killer

According to WHO [29], the disease burden attributable to air pollution is recognised as the single biggest environmental threat to human health and is estimated to be on a par with other major global health risks, such as unhealthy diet and tobacco smoking. Air pollution is considered the biggest environmental risk factor, i.e. for cardiovascular diseases, for type 2 diabetes, cancer and dementia, both of the Alzheimer type and the non-Alzheimer type [30–33] and thus a major driver of quality of life and survival.

Recent analyses estimate that at least around 7 million [34] premature deaths annually are due to fossil fuel pollution in households or elsewhere. Once again, low and middle-income countries are most affected [35], in particular in the Western Pacific Region, the South-East Asia Region and the Africa Region [36]. Thus, in terms of evidence-based policies, millions of lives could be saved, if knowledge were more widely available and alternatives provided. To give a very simple example: one of the major drivers of fine-particle derived cardiovascular diseases in Africa is cooking with wood and other solid biomass fuels [37, 38]. If such cooking were done with electrical sources, many of these patients may not have developed this disease. Simple measures can make a difference and evidence-based policies are needed to make this possible and to put knowledge into practice.

### Loss of biodiversity

There are other areas where we do not perform much research. One example is the extreme loss of biodiversity – some animal epidemiologists and researchers are speaking of a sixth mass extinction. We are losing species on an almost half-yearly basis [39]. At the same time, we rarely have an idea what this loss of biodiversity means in terms of more zoonotic diseases occurring, the impact on the food chain or what other diseases might evolve.

### Pollution of the oceans through plastic waste

With regards to plastic pollution in the ocean, not much research is being conducted either. There appears to be an almost exponential growth of (micro-) plastics in the ocean, but its impact is not yet comprehensively understood [40, 41].

Already today, there are an estimated 50–75 trillion pieces of plastic and microplastics in the oceans. Plastic waste makes up 80% of all marine pollution, endangering animals in and around the sea, and finding its way back to humans via the food chain. Microplastics have been found almost everywhere in drinking water, in salt, in the soil and even in beer [42]. Interestingly, this topic only became well-acknowledged in Germany and led to public outcry once it became apparent that microplastics had also found their way into beer. Everything else seemed to make little difference, but once it was clear that this affected beer, it became a political issue. So, if I were to need to address this issue in office, I would already know how best to lobby for an extensive solution.

### One health

In summary, we have to look at what we are doing by recognising that we find ourselves in a One Health situation: environmental health, human health and animal health are all interlinked. This is one of the reasons why I believe WHO is doing a wonderful job at focusing its activities on the One Health perspective.

## Current actions taken

Finally, I will give a number of examples of what we are doing in order to strengthen public-health policy-making, both internationally and nationally.

### International evidence-based policy-making during the German G7 presidency

With respect to the COVID-19 pandemic, we recognised very early on that there was very little pandemic control worldwide. Gaps and challenges became even more apparent, e.g. in numbers and training of staff, financial limitations, geographical distribution as well as the technical capacities and capabilities of public health institutions.

Therefore, at the G7, advised by a number of globally respected scientists, we pushed strongly for the creation of a pandemic fund at the level of the G20. Through this fund we will give money to countries, in particular poorer countries, to educate the workforce in pandemic control and to provide resources for surveillance in order to better detect outbreaks before they become pandemics. This pandemic fund, administered by the World Bank, came about as a result of evidence-based policy-making.

### National evidence-based policy-making during the pandemic

Similarly, when I entered office, we established an interdisciplinary and independent expert council at national level in order to ensure evidence-based policy-making during the COVID-19 pandemic. This included 16 very well-known researchers and experts in Germany: epidemiologists, clinicians, virologists and communication experts. For more than one year, they advised me on an almost bi-weekly basis. So, whenever I had a certain question, e.g. on an emerging COVID-variant, the best vaccination protocol or other aspects, I had exchanges with these experts and they advised me and the Chancellery directly. When necessary, they also gave written advice. In addition to relying on their expertise ourselves, we also made their expertise and their recommendations public.

### Government commission for a modern and needs-based hospital care

As outlined earlier, in this major hospital reform we are conducting, we wish to create a policy and payment environment where the best hospitals treat the most complex cases, with less of an incentive to follow the money, but rather to follow evidence-based guidance. For this, I have organised the government commission of 17 experts. They met more than 50 times and came up with a 50-page memorandum with recommendations, which I promised to follow. I want to continue to make good on the promise that I want to place evidence-based medicine and knowledge of epidemiology at the heart of policies and politics wherever possible.

Let me finish up with a key insight that I have gained over the last decades. I really think that epidemiology is a key science for addressing the problems that we are facing – and this is why I was so honoured to give the Cutter lecture in December 2022. Epidemiology is a key science for dealing with the consequences of population ageing, a key science for dealing with climate change, a key science for solving environmental aspects. Epidemiology, embedded into health policy-making, is essential in order to make a better world.
